# DOES A PERCEIVED CONNECTION TO A NEIGHBORHOOD REDUCE LONELINESS?

**DOI:** 10.1093/geroni/igz038.1963

**Published:** 2019-11-08

**Authors:** Kimberly J Johnson, Dolapo O Adeniji

**Affiliations:** 1 Indiana University-Purdue University Indianapolis, Indianapolis, Indiana, United States; 2 School of Social Work, Indiana Purdue University Indianapolis, Indianapolis, Indiana, United States

## Abstract

This study investigated whether perceived neighborhood quality was associated with chronic loneliness for adults 60 and older in the United States. Although loneliness can be episodic and overcome, chronic loneliness has been identified as a social determinant of health. Utilizing ecological systems theory we hypothesized that higher levels of neighborhood social cohesiveness would be associated with lower odds of chronic loneliness. We postulated that the networks available to people in the proximal area where they live could provide social opportunities for reducing loneliness. This idea was consistent with prior findings indicating the salience of neighborhoods for retirees, but inconsistent with research indicating the importance of a confidant in reducing loneliness. Data from the 2008 and 2012 Health and Retirement Study Psychosocial Surveys were used (n = 3530). Loneliness was measured using the 3-item scale developed by Hughes and colleagues in 2004. Findings from unadjusted logistic regression indicated that loneliness was inversely related to neighborhood cohesion as measured by an index of the trustworthiness, friendliness and helpfulness of neighbors and cleanliness, occupancy, lack of graffiti, and sense of belonging in the area (OR = .73, p < .001). When demographic and health-related factors were entered into the model the odds of being lonely were significantly lower for those with higher ratings of social cohesion (OR = .83, p < .001). These findings were consistent with the idea that neighborhoods are an important social place for older persons and interventions at the neighborhood level may be more effective than individualized treatment plans.

